# Emergency Lateral Canthotomy for Orbital Compartment Syndrome After a Motor Vehicle Collision at a Nontrauma Hospital

**DOI:** 10.7759/cureus.109510

**Published:** 2026-05-23

**Authors:** Nicholas Brotzman, Michael Coletta, Andrew Garfield

**Affiliations:** 1 Emergency Medicine, Inspira Health Network, Mullica Hill, USA; 2 Emergency Medicine, Inspira Medical Center, Mullica Hill, USA; 3 Ophthalmology, Cooper University Hospital, Camden, USA

**Keywords:** acute orbital compartment syndrome, blunt ocular trauma, canthotomy, emergency medicine training, emergency ophthalmology, orbital decompression

## Abstract

We present a case of a 60-year-old female patient who presented to a community hospital emergency department after a motor vehicle collision with car intrusion, with left eye proptosis, ecchymosis, decreased left-sided extraocular movement, and progressive eye pain. Clinical findings were consistent with orbital wall fracture and retrobulbar hematoma leading to orbital compartment syndrome (OCS). Lateral canthotomy was performed in the emergency department of an acute-care hospital without on-site ophthalmology prior to transfer to a tertiary care center. This case highlights the importance of recognizing OCS, which requires time-sensitive intervention to prevent permanent vision loss, and the ability of emergency physicians to perform the procedure in a suboptimal setting.

## Introduction

Orbital compartment syndrome (OCS) is a rare complication most commonly occurring after trauma that can be sight-threatening if not properly managed [1]. A retrospective analysis of all patients with an OCS at a level 1 trauma center between 2012 and 2015 found that the incidence of OCS among 18,093 patients with craniomaxillofacial emergencies after trauma was 0.088%, reflecting the infrequency of OCS [2]. Additionally, orbital hematoma, a common precursor to OCS, occurs in about 30% of patients with orbital fractures, but only 0.5%-1% of patients with orbital hematomas develop OCS [3]. For these reasons, immediate recognition, diagnosis, and efficacious surgical skills are required for management, especially in hospitals without an ophthalmologist or trauma service.

In OCS, an insult to the vasculature of the eye, whether the etiology is trauma, anticoagulation, or other causes, can lead to hemorrhage and therefore cause increased intraorbital pressure if untreated. This most commonly occurs in the setting of a retrobulbar hemorrhage. In these cases, the hemorrhage expands to fill the orbital cavity, leading to circumferential constriction of the vasculature and optic nerve, thus causing ischemia and potentially irreversible vision loss [4]. Because of the limited capacity of the orbital cavity to expand, hemorrhage and edema can rapidly cause OCS [5,6].

A highly skilled professional is critical in the acute management of OCS. Lateral canthotomy and cantholysis are preferred procedures for reducing intraorbital pressure and are ideally performed within two hours of injury [1,7]. In level I trauma centers, trauma surgeons and ophthalmologists are readily available when needed. In community or rural hospitals, this is not always the case. In these instances, lateral canthotomy and cantholysis are often performed by emergency medicine physicians, with survey data showing that only 38.9% of these physicians have seen a case and only 37.1% feel comfortable performing this surgery on their own [8]. Because of the rarity of OCS, the necessity for timely, effective surgical skills, and presentation in a community hospital, research on the management of such cases is warranted. We present a case of acute OCS with acute, effective surgical stabilization in a community hospital.

## Case presentation

A 60-year-old female patient presented to the emergency department after a motor vehicle collision during which the patient experienced a T-bone type accident, where she was the unrestrained driver and was hit on the passenger side door. The airbags were deployed with intrusion and totaling of her car. She denied losing consciousness; however, she presented with a significant left-sided periorbital hematoma and facial laceration, which she was unaware of. Following a complete primary and secondary survey, a focused eye exam was performed. The patient's initial intraocular pressures (IOPs) were 46 mmHg on the left and 23 mmHg on the right, measured with a handheld Tono-Pen (Reichert Technologies, Depew, NY). Visual fields were intact; however, left eye extraocular motion was significantly decreased globally. This raised significant concern for OCS. Immediate CT imaging was obtained.

Imaging and diagnostics

A CT of the maxillofacial bones revealed an acute, large, comminuted blowout fracture of the left orbital floor associated with extensive posttraumatic opacification of the left maxillary sinus. Additionally, there was a large amount of herniated left-sided intraconal fat into the left maxillary sinus, as well as the left inferior rectus muscle with concern for entrapment (Figure 1). More findings included an acute fracture of the left lamina papyracea (medial orbital wall), with minimal displacement, in addition to a left periorbital/facial soft tissue swelling/hematoma (Figure 2).

**Figure 1:**
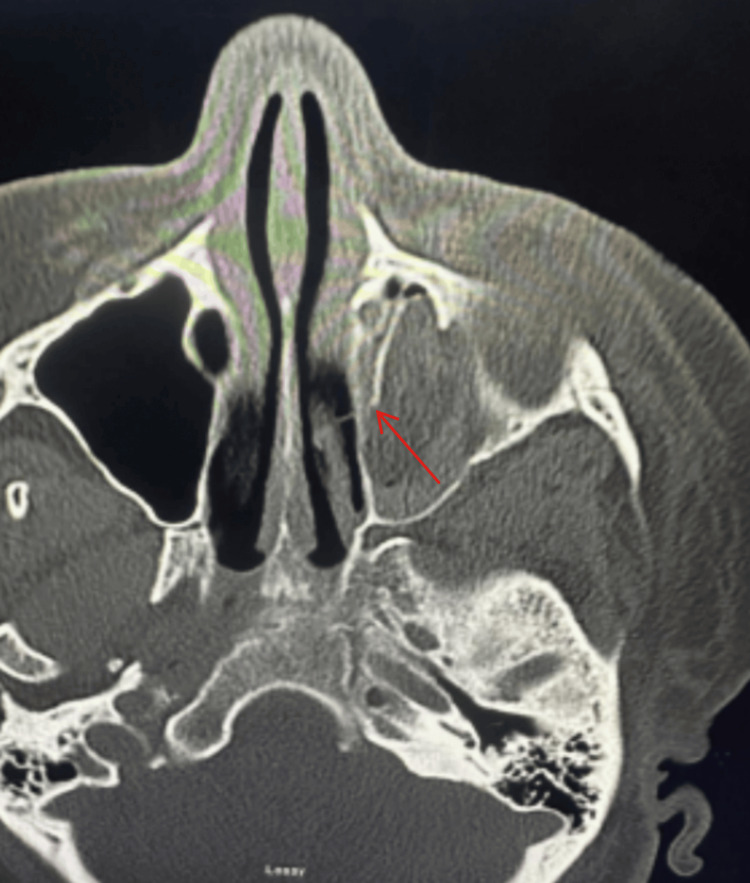
Axial view of maxillofacial CT imaging showing retrobulbar hematoma with entrapment of left inferior rectus muscle, consistent with OCS OCS: orbital compartment syndrome

**Figure 2:**
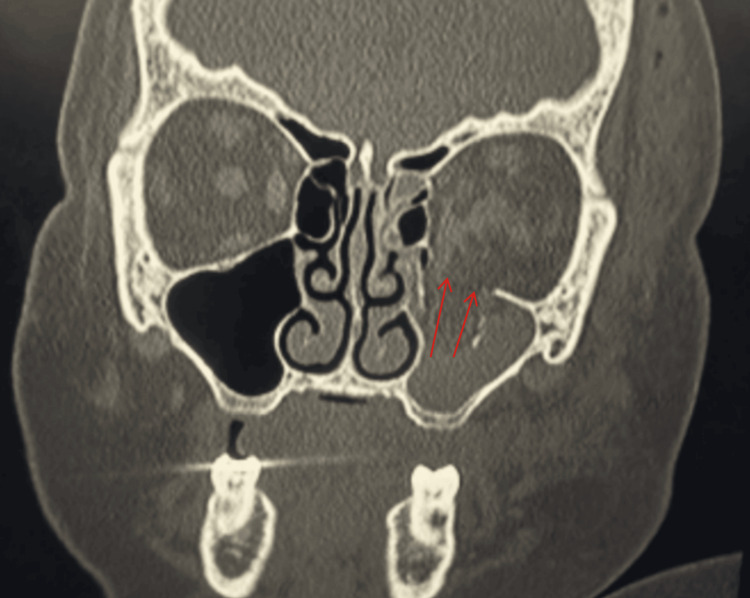
Coronal view of maxillofacial CT imaging showing inferior orbital wall blowout fracture and displaced medial wall fracture

Intervention and management

With significant exam findings of elevated IOP, decreased unilateral extraocular movement, and periorbital edema with ecchymosis, in conjunction with the imaging findings, OCS was diagnosed, and the decision was made to perform emergent lateral canthotomy at bedside in the emergency department prior to transfer, as the diagnosis and procedure were time sensitive to preserve vision. The patient was provided with fentanyl and midazolam for analgesia and anxiolysis. A left-sided lateral canthotomy was performed under local anesthesia with 1% lidocaine without epinephrine injected into the lateral canthus, which was then clamped with hemostats for approximately one minute. Sterile scissors were used to cut through the lateral canthus to the lateral orbital rim, using the canthal tendon as an anatomical marker. There was minimal blood loss, less than 10 cc overall. The patient experienced moderate pain during the procedure but tolerated it well, with some relief after the retrobulbar blood was expressed. Upon completion of the procedure, the transfer team entered the acute resuscitation room where it was performed to transport the patient to the tertiary care center. To avoid delaying care or transfer, the patient was emergently transferred for ophthalmology evaluation and management. On brief assessment just prior to patient transfer, extraocular motion was minimally improved, and the patient’s visual acuity remained intact prior to, during, and after the procedure.

Outcome and follow-up

No procedural complications were experienced. Upon completion of the procedure, transfer was available and ready, at which time the decision was made to forgo repeat imaging and IOP measurements, as the risks of delayed definitive treatment outweighed the benefits of repeat measurement. Posttransfer clinical course and long-term visual outcomes were not available, as the chart data from the accepting hospital were not accessible.

## Discussion

Diagnosis and management of OCS must be done in a timely manner. Delayed intervention may result in optic nerve ischemia and ultimately irreversible vision loss. Acute traumatic cases require a high clinical suspicion to ensure that management is done in an efficient manner. One study of 16 patients with OCS found that three (19%) patients developed permanent visual blindness, while another study found that 20% of patients developed no light perception [2,9]. Numerous studies and reviews suggest that interventions within two hours are associated with the best visual outcomes, with improvements in visual acuity after three hours being at just over half of patients. The latest intervention with improved visual outcome was at nine hours; however, this was in only one patient [9,10].

In this patient, the decision to follow through with lateral canthotomy was crucial. The community hospital where the procedure was performed does not have ophthalmology coverage and requires transfer to a trauma center for all patients who require immediate ophthalmologic evaluation. However, the time to intervention is crucial, and emergency medicine physicians are trained to identify OCS and perform a lateral canthotomy. Transfer delays would likely have worsened this patient’s condition, despite further ophthalmology resources at the transfer hospital. Ultimately, the decision for the emergency department staff to undergo lateral canthotomy was likely vision-saving for this patient and effectively prevented progression of OCS. The patient’s lack of decline in visual acuity and improvement in extraocular movements reflected success.

This case provides insight into the need for competent emergency medicine-trained physicians when handling OCS. Without procedural competence, despite minimal follow-up, specialists at this teaching hospital, in a time-critical condition like OCS, would have likely increased the risk of irreversible vision loss. Most emergency department physicians report having little to no experience in managing OCS, but the benefits of competent management may benefit many rural hospitals [8].

## Conclusions

Timely and competent management of OCS is crucial to prevent irreversible vision loss. In rural or community settings where specialists are less available, this is even more critical. The effective management of OCS of a 60-year-old patient following a motor vehicle accident evidenced by stabilization of visual acuity and improvement in extraocular muscles following lateral canthotomy at a community hospital proved the value of procedural competency and the increased need for physician training in management of OCS.
